# Guy's Hospital

**Published:** 1896-06-20

**Authors:** 


### The Institutional Workshop.

### GUY'S HOSPITAL.

Being actively interested in the success of the elaborate and wide-reaching organisation which resulted in making the festival at the Imperial Institute on the 10th inst. the most memorable of festivals in the cause of charity, we were a little disturbed by certain statements which were made to us as to the relative efficiency of the internal administration of Guy's compared with more modern hospitals in this country. We therefore paid a visit to Guy's, and, through the courtesy of Dr. Perry, the able and devoted superintendent, we spent six hours in going into every portion of the hospital, and examining closely the work done in all its aspects, and the conditions which prevail within the walls of Guy's to-day. Upwards of fifteen years have elapsed since we had an accurate knowledge of the interior economy of this institution, and it affords us genuine pleasure to state as the result of our searching investigations that we were pleased and surprised to find the enormous improvements which have been introduced throughout the establishment since Dr. Perry and Miss Nott-Bower, the present matron, have had charge of the management. Criticism has been passed on the fact that the £100,000 subscribed some years ago, although given as an endowment fund, has been spent upon maintenance. We are bound to say, however, that the point of this criticism largely fails when the facts are gone into and the results of this expenditure are realised and understood. Fifteen years ago Guy's lacked many things, and was far behind certain of the hospitals in the quality of its nursing staff, whilst the appliances were mostly old-fashioned and often insufficient. All that has now been altered, and, with the exception of the points to which we shall refer directly, no one who really understands hospital administration in all its bearings could fail to be satisfied with the internal arrangements of Guy's Hospital at the present time. The atmosphere of the wards is satisfactory. The present staff of sisters and nurses, encouraged by Miss Nott-Bower, have succeeded in giving every ward an air of comfort and homeliness which must be very grateful to the sick inmates. Everywhere there was evidence of devotion to duty, adequate precautions to maintain hygienic completeness, and nowhere did we see anything which the most fastidious could reasonably object to. We questioned some of the patients, and had the opportunity of conversing with the nurses and sisters, of examining the bedding, bedsteads, and various appliances, and understanding the system upon which each ward is administered. Everywhere we found the same evidence of thoroughness and progress, and it was a genuine pleasure to find that the slighting remarks made from time to time by ill-informed or thoughtless persons were without foundation or reason.

### An Opportunity for the Wealthy.

Of course, certain portions of the buildings are old, but in no case did we find that there was any occasion to find fault with the wards, though some of them might be improved by being refloored with teak, and others need to have the lavatories and bath-room accommodation enlarged and reconstructed. Here and there a ward contained more beds than we like to see, but the removal of a total of ten beds from the few wards referred to would overcome this objection, and make them as good for the treatment of disease as any modern wards which might be mentioned. Then, too, it would be an improvement, though it is not an absolute necessity, to replace some three hundred of the old bedsteads, and to substitute horse-hair mattresses for the flock beds which are still used at Guy's. Experience shows that in the hands of a trained nursing staff, where patients are got up for a certain time each day, and the flock beds are thoroughly shaken and aired, they prove to be comfortable to lie upon, and many of the objections which used to attach to them under a less thorough system have disappeared. Taking Guy's as a whole, we estimated that all the decorative and structural repairs and alterations, and the addition of new furniture and fittings so as to improve the whole institution and bring every portion of it up to date, would probably not entail a larger expenditure than £10,000. Here is an opportunity for a millionaire, who, by giving a cheque for £10,000 to be expended upon the existing buildings and in the replacement of old furniture and fittings, would for ever identify the donor with Guy's Hospital in the same way that the name of Hunt will ever be perpetuated by the buildings which are known as Hunt's House.

### Some Recent Improvements.

Bit by bit the whole of the sisters and the lady probationers' quarters have been redecorated, refurnished, and made wonderfully effective and pretty through the excellent taste manifested by Miss Nott-Bower, the matron. It is simply marvellous to see what a little thoughtful care and taste can effect in beautifying the interiors of old buildings and making them absolutely efficient as resident quarters for a large staff of well-educated women. Several new inventions of a highly valuable type have been utilised for the first time in the wards of Guy's Hospital; amongst them there is a new pattern of iron bedstead which, by a simple adjustment for the head, enables an excellent bed-rest to be made without difficulty or delay. This moveable bed-rest is also capable of being lowered, so that the patient may lie perfectly flat in the bed, and chloroform may be administered without difficulty or risk. Another excellent invention is the mackintosh rails, which might be introduced with advantage into every large general hospital. Much ingenuity has been expended in the design for these appliances, and we commend them to the study of all hospital managers throughout the country. The laundry-house, where the laundrymaids reside, has been adapted with much skill by the architect, Mr. J. H. Woodd, to whom much credit is due. We believe this to be quite a new departure in hospital economics, and it would be well if many institutions were either to imitate or improve upon it. It is a feature which might be adopted with advantage by similar institutions which do their washing on the premises. Nothing could be more excellent of its kind than this laundry-house, and the authorities of Guy's Hospital are to be congratulated for having instituted and placed it upon a footing of such marked efficiency.

The Nurses' Quarters.

The one feature which is at the present time unsatisfactory is the inadequate accommodation provided for the nurses. They are located in cubicles at the top of the ward blocks, and although much has been done to make them as good as circumstances will permit, they cannot continue much longer as they remain at present. The governors are fully alive to this fact, and contemplate, we understand, the erection of an adequate nurses' home, which should be replete with every modern contrivance, and of sufficient proportions to accommodate the whole of the nursing staff comfortably and properly. The site is available upon which the necessary buildings can be erected at a cost of from £10,000 to £15,000, and the sooner this necessary addition is made to Guy's Hospital the better. It is hoped that an excellent site for a nursing institution and midwifery school will be speedily available in the immediate neighbourhood of the hospital, and all friends of Guy's would rejoice to hear that the governors had been able to purchase such a site with the view of providing a modern building for housing the staff of nurses who are sent out to attend private cases. We have reason to believe that steps will be shortly taken which will secure excellent accommodation for the whole of the internal and external nursing staff attached to Guy's Hospital. In the course of our visit we were especially struck with the enthusiasm and the zeal of the sisters and nurses to which much of the marked improvement and the present notable efficiency of the internal administration is no doubt due. Everybody may give to Guy's with the assured confidence that all the money entrusted to the governors will be wisely utilised to promote the economical administration of one of our greatest and most valuable hospitals.

### THE DINNER.

Unique in the annals of charity dinners was the festival in aid of the re-endowment of Guy's Hospital, which was held on the 10th inst. at the Imperial Institute under the presidency of H.R.H. the Prince of Wales. The guests numbered 500, and were seated in groups of eight at small tables arranged in the new summer dining pavilion in the East Garden. This innovation succeeded most admirably, for not only was the general effect more pleasing than the usual arrangement of long tables ranged up and down the room, but, as far as possible, the opportunity had been given to those present to make up the parties seated at each table. The Prince of Wales was received at twenty minutes past seven o'clock at the east entrance by a reception committee, and was conducted to the dining pavilion, where the guests were already assembled.

After Dinner

The Prince of Wales, who was greeted with loud cheers, having briefly proposed the toast of "The Queen,"

The Right Hon. A. J. Balfour, M.P., proposed "The Prince and Princess of Wales and the rest of the Royal Family." In doing so he said that his Royal Highness had ever given his powerful assistance to great philanthropic and benevolent causes, but he doubted whether it had ever been his fortune to give his assistance to a worthier cause or on a more important occasion than the present. (Cheers.) He reminded those present that they had not merely assembled in order to support a great charity—the well-to-do giving to the needs of those who are less well-to-do. That was a great aspect, perhaps the principal aspect of this question of Guy's Hospital; but there was another aspect, and that was this: that, of all forms of human energy and all aspects of human progression, that which touched upon medical and surgical science went most straight and direct to the diminution of human pain, and to the increase of human happiness, and that if people were to make all the progress which was possible in the medical and surgical science of the future, great medical schools were an absolute necessity. Let them remember, therefore, that Guy's Hospital was one of the greatest medical and surgical schools in the country. (Cheers.) It ministered not to one class alone, but to the whole community, and to future generations—to generations yet unborn. (Hear, hear.) That night, therefore, they were not merely aiding and assisting to the best of their ability medical and surgical relief for the poor, but they were doing something to advance medical and surgical science, and in so doing they were undoubtedly contributing largely to the general welfare of the whole community. (Applause.)

The toast having been drunk amid loud cheers,

The Prince of Wales replied, cordially thanking those present for the enthusiasm with which they had received the toast. The very large gathering that night proved to him the great interest all took in the subject before them. It would be difficult—he might even say impossible—for him to return individual thanks to all who had so kindly supported him that evening, and who had come there in such numbers to give their countenance and support to the great scheme they had before them. He knew that the members of the hospital and its staff had worked very hard to accomplish what had been done—(applause)—and he might be allowed to mention the name of a personal friend of his who had worked so hard, namely, Mr. Fripp. (Cheers). He must also not forget one of the greatest philanthropists whose name was so well known to all, who, in the cause of charity, and especially of hospital work, had done so much, and to whom they were indebted for the trouble he had taken in making that hospital dinner a success. He alluded to Mr. Henry Burdett. (Cheers.) As showing the great interest taken in the proceedings that night, he mentioned that a telegram had been received from Captain Lugard, in South Africa, with whom, as a medical officer, was an old Guy's man, Mr. Spon. (Cheers.) His Royal Highness then proposed "The Navy, the Army, and the Reserve Forces."

The Hon. Sir Harry Keppel, G.C.B., Admiral of the Fleet, having responded for the Navy,

Field-Marshal Lord Roberts, V.C., G.C.B., replied for the Army. In the course of his speech he said that there was a closer connection than might at first sight appear between the Army and Reserve Forces and the London hospitals, amongst which Guy's Hospital occupied such a prominent place. In the scheme for the defence of the United Kingdom, provision had been made in London for the reception and treatment of 20,000 sick and wounded soldiers; the chief metropolitan hospitals had undertaken to look after 3,000, and of this number Guy's Hospital had arranged to take 800. (Applause.) Looking at the question, therefore, not only from a humane point of view, but from that of expediency, the country could not afford to see such a useful institution seriously crippled, or perhaps pass out of existence for want of funds. (Applause.)

"Guy's Hospital."

His Royal Highness the Chairman, in proposing the toast of the evening, said: We have met here this evening to take such steps that the benevolent work of Guy's Hospital, which for so long was carried out on a magnificent scale without the aid of one shilling from the general public, may be continued to an extent not more contracted than hitherto. I may add that this long exemption from the necessity for an appeal made it very difficult for the authorities to remove the popular impression that Guy's is as permanently and as sufficiently endowed as other national institutions which bear the same world-wide reputation. The founder, Thomas Guy, a citizen of London, and a bookseller and publisher, invested his money so that for one hundred and fifty years the income derived from it was quite sufficient to carry on the great work he had devised. No assistance was asked, indeed, none was required, and the governors were in a position to inform intending donors of a fact which, I fear, institutions are rarely able to assert in these days, that there were other charities in greater need than Guy's. It was in 1724 that Guy founded the hospital which bears his name. The population of South London, as of every other part of London, has steadily increased, and a very large percentage of its inhabitants are extremely poor. One hundred years later—in 1822—a second merchant and citizen of London, William Hunt, left £180,000 to enable the original foundation to support the increased demands upon it. Guy's building is now used entirely as surgical wards, and the block known as "Hunt's House" is wholly devoted to medical cases. During the next fifty years—1830 to 1880—the hospital was able to maintain its wards, comprising 600 beds, fully equipped and always crowded, but adequately endowed. At last, however, fifteen years ago, there occurred the great fall in the value of land, in which, according to the will of the founder the entire capital bequeathed had been compulsorily invested. Then, for the first time, the endowment proved insufficient. The governors, hoping the depression was only temporary, closed 100 beds. This decline in the value of land not only continued, but increased, and the governors, brought face to face with the necessity of still further curtailing the scope of the hospital, or of enlisting support, appealed for £100,000 to carry them over what they still hoped was only a temporary difficulty. £114,000 was generously given, but has all, unfortunately, been expended, for notwithstanding that 100 beds have been closed and that no hospital is more economically managed, £40,000 a year is required to maintain this great charity, whereas the endowment now only brings in £20,000 per annum. We must now, unfortunately, recognise the fact that the depression has proved to be not merely temporary, but permanent, and we have met here to night to decide whether the board of governors, who are again in the same dilemma, can continue to carry on the work of this noble institution with its former thorough efficiency without curtailment of its previous extensive sphere of action. Owing to its position, on the outskirts of a densely populated and poor district, it has been called upon to minister to the requirements of no fewer than 160,000 patients a year. Every day we have been compelled to turn away cases from the doors, and to send them on a tedious, sometimes a painful, journey through the London streets to some less crowded hospital. If any of these whom I meet here to-night would go to Guy's—and I speak from personal experience—(loud applause)—having had the opportunity about a month ago of visiting it—and see for themselves the overcrowded state of the wards and the disappointed faces of these for whom beds cannot be provided, they would, I am sure, agree with me that it would be deplorable were the authorities compelled to close still more beds. (Hear, hear.) If we are unable to act on the recommendation of the House of Lords' Commission, and to establish a third great general hospital in South London for the poor, we may, at all events, strive to strengthen the endowments of our forefathers, so that the funds which result from them may not be impaired, since we succeeded to the inheritance. To achieve this in the case of Guy's, a large sum, it is true, is required, but when I think of the generosity with which the people of England so often come forward in aid of any really good cause, I cannot but hope that the amount necessary, or, at least the greater part of it, will be forthcoming. The sum necessary for the purpose is £15,000 a year, or, if capitalised, half-a-million of money. It is hardly necessary for me to enter in detail into the progress in surgical and medical science, which has conferred such great distinction on Guy's, and which I earnestly beg you to remember has proved such an incalculable blessing not only to the poor suffering in the wards, but to humanity generally. I may, however, mention to you the names of Dr. Bright, who discovered in the wards at Guy's the origin of the terrible disease which bears his name; of Sir Astley Cooper—(loud cheers)—perhaps the most eminent surgeon of the old school whom England has ever produced; of Dr. Hodgkin, of Sir William Gull, to whom I personally owe a debt I can never forget, for I think had it not been for him I should not have had the honour of presiding here to-night—(cheers)—and last, but not least, I allude to Dr. Wilks, the president of the Royal College of Physicians. (Cheers.) Of the many classes who are so generously responding to this appeal, few, if any, have contributed more liberally in accordance with their means than the former graduates of Guy's—(applause)—who, in all parts of the world, have so actively co-operated with their old teacher and friend, Dr. Wilks. (Loud applause.) My lords and gentlemen, I am anxious to take up as little of your time as possible in pressing any further the claims of Guy's, but I cannot sit down without referring to its admirably economical administration and its high degree of efficiency. To these features testimony is borne by the senior governor, Mr. Gladstone—(applause)—whose unavoidable absence to-night we all so much regret, and by many others who have made it their business to investigate them. We have a hard-working and well-directed staff available, but money is required to utilise it. If this deficiency were supplied, we could at once open every one of the 140 beds which are so urgently needed, which are ready for occupation, and yet which we are compelled to keep closed simply through mere want of funds to feed the patients and to provide the nursing. Were the case otherwise, small outpost hospitals could then be established in the most densely crowded of the outlying districts, with a good ambulance service to bring them into easy communication with Guy's, and thus we should save the cost of separate establishments, whilst we should retain the advantage of providing the patients with the highest medical and surgical skill. The generous promises which have been made in response to the special appeal to the south-east district, show, I think, how thoroughly the few residents who are able to give appreciate the work done by Guy's for the many. Once more I repeat—money remains our one indispensable requirement. For reasons which I have already indicated, our accounts do not show that thus far we have derived any benefit from legacies. But we hope in the future many benevolent testators will not be forgetful of the merits and needs of Guy's Hospital. (Applause.) I trust, in addition, that through donations to this special endowment fund, and through increased annual subscriptions, a sufficiently large sum will be forthcoming to render practicable shortly the extensions to the building, and the further development of administration to which I have already referred. Many who cannot afford a large donation at present in a single sum might perhaps be willing to supplement what they are now able to give by the promise of a larger amount distributed over three or five years; I would ask those who are kindly inclined to inscribe their names as donors to be good enough to intimate to the secretary whether they desire to pay their entire contributions at once or to spread them over a period of, say, three or four years. In conclusion, just let me remind you that the whole of the money given in response to this appeal will be invested by the strong committee of well-known financiers whose names have been published in the public press, and that only the interest derived from the investments will be expended. The money already subscribed, and which is large enough to make this evening for ever memorable in the annals of charity dinners, amounts to £158,000—(loud cheers)—which, capitalised, would bring in an increased annual income of £5,000. It remains only now for me to propose to you the toast of the evening, which is "Prosperity to Guy's Hospital"—(cheers)—and may it long continue to be successful and prosperous. (Renewed cheers.)

The toast was drunk upstanding, three cheers being given for Mr. Lushington.

Mr. E. H. Lushington said that the duty of replying to that toast had fallen upon him in consequence of the illness of the president of the hospital, Lord Aldenham. Each year new streets were being added to the East of London, and as the houses became occupied so followed diphtheria, typhoid, and all those various accidents to which flesh was heir, against which that hospital had to endeavour to supply assistance. Hitherto they had talked a good deal about that magnificent hospital having been maintained at the sole cost of the late Thomas Guy. He regretted that they could no longer refer to that as applying at the present time. They must look back upon it as a monument of the noble generosity of a single individual citizen of London, and they must henceforth refer to it as a living testimony of the same public beneficence and public liberality which had so helped them on the present occasion. (Applause.) The fact of their being so largely supported by the public would involve on their part some modification of the laws and rules which had hitherto regulated the government of the hospital. The governors were prepared to consider what modifications might seem advisable with great caution and deliberation. He hoped to be spared to take part in that duty, and when those matters had been fully considered and had met with the approval of their president, he had then to say that the time had come when they must find another treasurer. Twenty years of work, he thought, seemed sufficient, inasmuch as it was preceded by many years of public work before.

"The Medical School."

His Royal Highness the Chairman, in proposing the toast of "Guy's Hospital Medical School," said that the connection between the medical school and the hospital was necessarily of a most intimate character, and it was their co-operation which had in the past established the high reputation of the Guy's School as a training ground for medical men, and continued to furnish them with the surest guarantee that that reputation would be maintained unimpaired in the future. His Royal Highness, in expressing the great gratification it had afforded him to accede to the request of the governors that he would accept the post of president of this great hospital—(loud cheers)—confessed that he felt great diffidence in acceping so important a post; but all those connected with the hospital might feel assured that to the utmost of his ability he would render what poor service he could for its advancement. (Renewed cheers.) He felt that he was succeeding one to whom the hospital owed much—(hear, hear)—and after his fifteen years of office his loss would, he knew, be keenly felt; but Lord Aldenham felt from ill-health that he could no longer continue to occupy the position, and he was sure it was with sincere regret that they did not see him amongst them that night. When he told them the reason Lord Aldenham would he was sure have their hearty sympathy. Just before dinner he (the Prince of Wales) had a telegram stating that the principal reason for his absence was the dangerous, if not the dying, condition of his wife. His Royal Highness regretted that Mr. Lushington, who had for twenty years occupied the high and difficult post of treasurer, also felt, after so long a time, it more prudent to resign that post. He also allude to Dr. Perry—(loud cheers)—a man who had done so much for the maintenance of the hospital as superintendent; and last, though not least, to the matron. (Hear, hear.) They were all well aware what a high and responsible position she held, and however adequate and clever the medical staff might be, unless the nurses did their duty it was difficult for them to carry out all that science had taught them.

A total of £8,524 subscribed at the banquet was then announced, including £5,000 from Mr. J. B. Robinson.

Dr. S. Wilks, F.R.S. (President of the Royal College of Physicians, who is also consulting physician to Guy's Hospital), responded. In the course of an excellent speech he said that as His Royal Highness had stated his intention of becoming president, he begged leave to thank him in the name of all those connected with the hospital for that most recent gracious act of his. (Loud cheers.) When at the beginning of the year the hospital was in great straits, they turned to the Prince of Wales for aid, and their appeal was not made in vain. Owing, not only to his influence, but to his own personal efforts, sunshine had appeared where before all was darkness, and now they had a grand vision of rehabiliment before them. In the history of their country no one stood out with more prominence as a friend of mankind than Thomas Guy, their founder, and now, future history would show how, when his great gift was in fear of loss or decay, the great hospital was re-established and re-endowed under His Royal Highness's auspices, and that day would be remembered as its second birthday. (Applause.) On behalf of the governors, of the medical staff and teachers, he (Dr. Wilks) thanked the Prince again and again for that gracious act of becoming their president, and taking them under his fostering care. (Loud cheers.)

Details of the Amount Collected.

The following shows how the large sum received in response to the appeal of the Prince of Wales, and announced during the dinner, is made up:—

| | |
|---|---|
| Collected and subscribed by the medical and surgical staff and the past and present students of Guy's Hospital Medical School ... ... ... ... | £26,510 |
| Subscribed by members of the Stock Exchange ... | 10,606 |
| First instalment of collection in progress among the members of the Textile and Furnishing Trades of London ... ... ... ... ... ... ... | 7,000 |
| Subscribed by members of the Commercial Sale Rooms and their friends ... ... ... ... | 2,629 |
| Racing Fund ... ... ... ... ... ... ... | 1,559 |
| Subscribed by members of the Leather Trade ... | 927 |
| Subscribed by members of "The Baltic" ... ... | 498 |
| Subscribed by members of the Corn Exchange ... | 273 |
| First instalment of collection in progress on the Shipping Exchange ... ... ... ... ... ... | 175 |
| Individual donations ... ... ... ... ... | 55,646 |
| Subscriptions and donations received for the Sustentation Fund, principally in response to the appeal of the Right Hon. W. E. Gladstone on October 30th, 1895 ... ... ... ... ... ... | 18,181 |
| Residue of the Estate of the late Mr. F. G. Incleden, of Falmouth, Christmas, 1895 ... ... ... | 35,000 |
| Subscribed at the Banquet ... ... ... ... | 8,524 |
| Total ... ... | £167,528 |

### THE RECEPTION.

Even before the appointed hour for the reception streams of carriages wended their way towards the Imperial Institute. By a quarter-past nine locomotion in the passage ways of the vestibule had become a difficult matter, and the presence of the police was of great assistance in circulating the crowd, all eager to obtain the best point of view to see the Prince on leaving the banquet pavilion. That the "coming doctor" took the keenest interest in the occasion there could be no question, for the contingent of students, accompanied by cousins and aunts, formed a large element in the company. The aspect of anticipation which was noticeable among the guests made the gathering unlike the usual assemblages of London folk, drawn together through motives of expediency rather than expectation of pleasure. The presence of the Prince was obviously the feature of the night, and as the promised entertainments on the programme all hung on his appearance after the banquet, the impatience with which his presence was awaited was very marked, and his appearance was hailed with great enthusiasm shortly before eleven o'clock. The geography of the institute was obviously unfamiliar to a large number of those present, and the police found their occupation as animated signposts more arduous even than in the streets. "Where is the Prince coming from?" "Where can we go?" "Where are the motor cars?" was asked ceaselessly throughout the evening; whilst one of the frankest of the gathering made for the nearest policeman on arriving, and was heard to ask, "Kindly direct me to the refreshments, I want to make a bee line for them in good time," which he effectually did by means of a little determined and vigorous pushing. The refreshments, by the way, were plentiful and well managed. The entertainments consisted of the music of the Monte Carlo Band, the motor cars, which ran under very distinguished patronage, and the X Ray photography. Thither the Prince repaired after the conclusion of the dinner, and a very successful photograph of his hand was taken. The Reception Committee repaired to the band pavilion, where they were more evident to the assembled company than on arrival, when there was far too great a crowd to distinguish anyone. Amongst the committee were the Duchess of Portland, the Duchess of Marlborough, Countess Grey, the Countess of Warwick, and the Marchioness of Londonderry. The evening was far from warm, and so airy were the corridors that those ladies who had retained their wraps were deemed very fortunate. The rain kept off, but one gentleman had cautiously provided himself with an umbrella, which he retained during the evening, oblivious of the amusement it caused. The inconvenient crowding which was experienced by the 4,000 or 5,000 guests assembled was almost welcomed, as it was felt the more the better for the sake of the hospital.

